# Genotypic and phenotypic characterization of resistance to fenhexamid, carboxin, and, prochloraz, in *Botrytis cinerea* isolates collected from cut roses in Colombia

**DOI:** 10.3389/fmicb.2024.1378597

**Published:** 2024-07-31

**Authors:** Diego Giraldo, Catalina Saldarriaga, Héctor García, Miguel López, Adriana González

**Affiliations:** ^1^Facultad de Ciencias Agrarias, Universidad Nacional de Colombia, Sede Bogotá, Bogotá, Colombia; ^2^Laboratorios Diagnofruit Chile, Santiago, Chile; ^3^Laboratorios Diagnofruit Colombia, Cajicá, Colombia

**Keywords:** phylogeny, *Botrytis*, resistance, fungicide, floriculture

## Abstract

Gray mold, caused by *Botrytis* sp., is a significant disease in Colombian rose crops and its control depends primarily on the intensive use of chemically synthesized fungicides. Despite the importance of this pathogen, there is limited information in Colombian floriculture about molecular taxonomy of species, fungicide resistance of populations and their genetic mechanism of resistance. In this study, we analyze 12 isolates of this fungus collected from rose-producing crops in the Department of Cundinamarca and conducted phylogenetic analysis using *HSP60*, *G3PDH*, and *RPB2* gene sequences. Additionally, we realize phenotypic and genotypic characterization of resistance to the fungicides fenhexamid, carboxin, and prochloraz, evaluating the *in vitro* EC_50_ and presence of mutations of target genes of each isolate. All isolates were characterized as *Botrytis cinerea* in the phylogenetic analysis and presents different levels of resistance to each fungicide. These levels are related to mutations in target genes, with predominancy of L195F and L400F in the *ERG27* gene to fenhexamid resistance, H272R/Y in the *SDHB* gene for carboxin resistance, and Y136F in the *CYP51* gene for prochloraz resistance. Finally, these mutations were not related to morphological changes. Collectively, this knowledge, presented for the first time to the Colombian floriculture, contribute to a better understanding of the genetic diversity and population of *B. cinerea* from rose-producing crops in the department of Cundinamarca, and serve as a valuable tool for making informed decisions regarding disease management, future research, and improving crop management and sustainability in the Colombian floriculture industry.

## Introduction

Colombia is classified as a primary global producer and exporter of cut flowers, with rose cultivation (Rosa x *Hybrida* L.) being the most representative (Buitrago, 2021). However, the productivity and quality of flowers can be affected by various diseases and pests, such as gray mold caused by *Botrytis* sp., which is one of the most devastating diseases in rose-producing crops. This disease leads to significant economic losses owing to a reduction in both the quality and quantity of harvested flowers (Muñoz et al., 2019). In ornamental crops, the disease can manifest in different plant tissues, such as leaves and stems; however, the most severe economic damage occurs when the pathogen infects flowers (Muñoz et al., 2019). Initial symptoms in this type of tissue appear as small spots that progress to necrotic tissue, leading to the collapse of petals and the floral bud (Fillinger et al., 2016). This disease causes annual losses ranging from 10 to 100 billion dollars worldwide (Elad et al., 2016; Garfinkel, 2021).

The genus *Botrytis* encompasses various species, with *B. cinerea* being one of the most generalists, capable of infecting more than 1,400 plant species in almost 600 genera (Garfinkel, 2021). Traditionally, the identification of the *Botrytis* genus has relied on the observation and differentiation of morphological characteristics such as the size and shape of conidia and conidiophores, the production, organization, and size of sclerotia, and the morphology of colonies in culture media (Walker, 2016). However, these methodologies may have limitations because of the variability in observed characteristics and the difficulty in distinguishing between closely related species (Garfinkel, 2021). In addition to morphological classification, molecular classification has been used for the *Botrytis* genus based on the analysis of genetic composition and phylogenetic relationships between species. This technique involves the sequencing of multiple regions of DNA in the genome of an organism, typically focusing on conserved and highly informative genes to obtain more complete information about its genetic diversity (Staats et al., 2005). Among these genes are those encoding heat shock protein 60 (*HSP60*), glyceraldehyde-3-phosphate dehydrogenase (*G3PDH*), and RNA polymerase subunit II (*RPB2*) (Walker, 2016). This approach has facilitated the most recent classification of the genus into 38 species that are distributed across three clades (Garfinkel, 2021). Additionally, these genes play a crucial role in the study of phylogenetic relationships, genetic diversity, and species differentiation (Walker et al., 2011). However, a multilocus sequence analysis (MLSA) of housekeeping genes of *Botrytis* species that affect floriculture in Colombia has not yet been explored.

Although integrated grey mold management strategies include beneficial microorganisms such as *Bacillus* sp., protective and systemic fungicides remain part of disease management (Samaras et al., 2016; Hashem et al., 2019). In Colombia, the commonly used fungicides in rose crops belong to the FRAC codes 1, 7, 9, 10, 12, 17, M03, and M04, and the frequency of systemic fungicide application can reach up to three times a week, depending on the incidence of the disease. These fungicides can act on a specific site of the physiological processes of the fungus or can be multisite, possessing several action sites simultaneously (Hahn, 2014). Additionally, some of them are used to control other pathogens such as *Podosphaera pannosa* (powdery mildew). However, the high frequency of their applications implies intense selective pressure combined with the adaptive plasticity of *Botrytis*, which has led to a decrease in the sensitivity of the fungus to various fungicides and the absence of growth inhibition *in vitro* at lethal concentrations (Gisi and Sierotzki, 2008). Similar trends of the accumulation of multiple resistance in *B. cinerea* have been recently reported for stone fruits, tree seedlings such as raspberries, strawberries, grapes, and ornamental flowers in Germany and Brazil (Rupp et al., 2017; Ishii, 2023).The fungicides commonly used for controlling gray mold in rose crops of Cundinamarca are fenhexamid, carboxin, and prochloraz, all of which affect essential mechanisms for the development of the fungus. fenhexamid inhibits the enzyme 3-keto sterol reductase, encoded by the *ERG27* gene, and catalyzes the demethylation of C4 during ergosterol biosynthesis (Debieu et al., 2001). On the other hand, carboxin is a fungicide that belongs to the succinate dehydrogenase inhibitors (SDHI) group, a key protein in the cellular respiration process (Avenot et al., 2009); and prochloraz, acts by inhibiting the enzyme sterol 14α-demethylase, encoded by the *CYP51* gene and related to cytochrome P450 superfamily (Zhang et al., 2020).

The increased prevalence of multiple resistance in *Botrytis* populations has limited the options for implementing strategies as rotation or mixture of fungicides for disease control and fungicide resistance management (Shao et al., 2021). Therefore, knowledge and monitoring of the sensitivity and development of resistance of this fungus to fungicides used for its control are crucial factors to consider in disease management and the search for integrated strategies. This study aimed to characterize morphologically, molecularly, and phylogenetically *Botrytis* isolates obtained from rose-producing crops in the Department of Cundinamarca, as well as to characterize their phenotypic and genotypic resistance to fenhexamid, carboxin, and prochloraz, the fungicides commonly used for controlling gray mold in this crops.

## Materials and methods

### Plant material and pathogen isolation

For the recollection of plant material, three rose-producing crops located in Tocancipá (CP), El Rosal (ML), and Madrid (MO) in the Department of Cundinamarca, Colombia, were chosen due to their high frequency of fungicide application (twice a week) for gray mold control. From each crop, a greenhouse with roses (Rosa *× hybrida* L.) of the Orange Crush variety was selected. In each greenhouse, five points were selected, and from each of these, four random samples were taken. Each sample consisted of three flowers showing initial symptoms of the disease, which were then placed in a humidity chamber until mycelium of *Botrytis* was observed.

Subsequently, *Botrytis* isolation was performed on PDA medium, following the methodology described by Avenot et al. (2020). Once purified, the isolates were subjected to monosporic culture, using the methodology described by Li et al. (2012). A monosporic isolate of *Botrytis* named NT was obtained from wild rose flowers of an unidentified variety, not subjected to fungicide management, and located in the greenhouses of the Faculty of Agricultural Sciences (FAC) of Universidad Nacional de Colombia, Bogotá campus. The NT isolate was used as a control strain, which was not exposed to the fungicides used in this study. Additionally, *B. cinerea* strain B05.10, which is a commonly used wild-type strain (Fillinger et al., 2008; Veloukas et al., 2011; Staats and van Kan, 2012; Leisen et al., 2022; Klug et al., 2024) was used as a sensitive reference isolate and was kindly provided by Dr. Cecilia Ramos of Nucleus of Applied Research in Veterinary and Agronomic Sciences, Universidad de las Américas, Chile.

### Macroscopic and microscopic characterization of isolates obtained from roses

The evaluation of the macroscopic characteristics of each fungal colony were conducted after 7 days of growth on PDA plates under dark conditions. Aspects such as color according to the Pantone^®^ scale (Pantone Color palettes Pantone EMEA),^1^ appearance, and presence of sclerotia formation were considered. The sclerotia formation was determined when the mycelium generated white or gray hyphae aggregated in small knots (Muñoz et al., 2019).

To perform microscopic characterization of each colony, microcultures were generated for each isolate. The presence of sporulation was assessed after 7 days of incubation, determined as the moment when the first conidiophores with conidia were observed under a microscope (Muñoz et al., 2019). Images of the microcultures were analyzed under a microscope at 400X using the ZEN lite software.

### DNA extraction and PCR amplification for conserved genes for phylogenetic analysis

Each monosporic isolate of *Botrytis* was grown in 30 mL Sabouraud Dextrose Broth (Scharlab), to which mycelium fragments from each isolate were added. Each tube was incubated for 3 days at 18°C with agitation at 80 rpm. For DNA extraction, the mycelium from each culture was macerated with the aid of liquid nitrogen, following the protocol of Pryor and Gilbertson (2000). The integrity and purity of each DNA sample was verified by agarose gel electrophoresis at 1.5% stained with ethidium bromide. *HSP60*, *G3PDH*, and *RPB2* genes were PCR-amplified using the primers detailed in Table 1. The amplification reactions were carried out in a final volume of 20 μL containing 1X Polymerase buffer, 2 mM MgCl2, 0.2 mM dNTPs, 0.2 μM of each primer, 1 U of PlatinumTM Taq DNA Polymerase (Invitrogen), and 2 μL of DNA. Amplification of the *HSP60* and *RPB2* genes was conducted with the following thermal profile: 94°C for 5 min, followed by 35 cycles of 94°C for 30 s, 55°C for 30 s, and 72°C for 90 s, and finally an extension of 72°C for 10 min. For the *G3PDH* gene, the amplification conditions were 94°C for 5 min, followed by 35 cycles of 94°C for 30 s, 55°C for 30 s, and 64°C for 90 s, and finally an extension of 72°C for 10 min (Staats et al., 2005). The products obtained from each amplification were evaluated using 1.5% agarose gel electrophoresis at 80 V.

**Table 1:** Primers used to amplify the *G3PDH*, *HSP60*, *RPB2* genes.

Gene	Primer name	Sequence (Sense 5′-3′)	Fragment size	Reference
*G3PDH*	BcGPD-F	ATTGACATCGTCGCTGTCAACGA	790 pb	Staats et al. (2005)
	BcGPD-R	ACCCCACTCGTTGTCGTACCA		
*HSP60*	BcHSP60-F	CAACAATTGAGATTTGCCCACAAG	1769 pb	
	BcHSP60-R	GATGGATCCAGTGGTACCGAGCAT		
*RPB2*	BcRPB2-F	GATGATCGAGATCATTTCGG	1750 pb	
	BcRPB2-R	CCCATAGCTTGTTTACCCAT		

The amplicons were sequenced at Macrogen Inc., Korea using the Sanger methodology with the primers listed in Table 1. The resulting sequences were analyzed using CLC Main Workbench v5.5 (CLCBio), and the consensus sequences were aligned with the NCBI database using the BLASTn algorithm (Basic Local Alignment Search Tool).^2^ The parameters considered included an E-value equal to or less than zero, a percentage of identity greater than 99%, and query coverage of 100%.

### Phylogenetic analysis

The sequences of the *G3PDH*, *HSP60*, and *RPB2* genes from each isolate were aligned using the ClustalW algorithm in the CLC Main Workbench v5.5 program (CLCBio). To represent the diversity of species within the genus, 83 sequences from 24 *Botrytis* species were included and selected according to Staats et al. (2005), Azevedo et al. (2020), and Notte et al. (2021), all of them available in GenBank (Supplementary Table S1). The sequences of *Sclerotinia sclerotiorum* and *Monilinia fructigena* were used as outgroups (Staats et al., 2005). Poorly aligned positions and divergent regions in the sequence alignment were removed using Gblocks. The maximum likelihood (ML) method was used to construct the phylogenetic tree, along with the Kimura evolution model with Gamma distribution (K2 + G), considering 1.000 bootstrap replicates. Phylogenetic analyses were conducted for each of the three genes (*RPB2*, *HSP60*, and *G3PDH*) and a concatenated matrix of the three genes. Both the evolution model and tree generation were determined using the MEGA v.10 software.

### Determination of the mean effective concentration (EC50) of fungicides

To assess the mean effective concentration of fungicides on *Botrytis* isolates, the culture medium was supplemented with the fungicides carboxin, prochloraz, and fenhexamid at analytical grade (> 98% purity of the active ingredient, Sigma Aldrich). carboxin was incorporated into PDA agar (Condalab), prochloraz into malt extract agar (Condalab), and fenhexamid into Sisler medium (0.2% KH_2_PO_4_, 0.15% K_2_HPO_4_, 0.1% (NH_4_)_2_SO_4_, 0.05% MgSO_4_·7H_2_O, 1% glucose, 0.2% yeast extract, and 1.25% bacteriological agar) (Weber et al., 2011). Final doses of 0, 0.01, 0.1, 1, 10, and 100 ppm for carboxin; 0, 0.02, 0.05, 0.1, 0.5, 1, 5, and 10 ppm for prochloraz; and 0, 0.03, 0.1, 3, 10, and 25 ppm for fenhexamid were prepared. Each supplemented medium was inoculated in the center of a Petri dish with a 5.5 mm fragment of each *Botrytis* isolate. This process was performed in triplicate. After three days of incubation at 25°C in the dark, the diameter of each colony exposed to each concentration was measured in two perpendicular directions (Avenot et al., 2020). The inhibition percentage of each dose of the evaluated fungicides was determined using the following equation (Panebianco et al., 2015):

$$\%\mathrm{inhibition}=1-\left(\frac{\mathrm{Isolate}\;\mathrm{growth}\;\mathrm{with}\;\mathrm{fungicide}\;\left(\mathrm{mm}\right)\;}{\mathrm{Isolate}\;\mathrm{growth}\;\mathrm{at}\;0\;\mathrm{ppm}\;\left(\mathrm{mm}\right)}\right)*100.$$

The EC_50_ value was calculated for each isolate and fungicide, representing the percentage inhibition of growth against the log_10_ of fungicide concentrations in the three replicates. The values were subjected to Probit analysis using Minitab^®^ V12 software (Veloukas et al., 2011). Additionally, the resistance factor (RF) of each isolate was calculated by dividing the EC50 value by the mean EC_50_ value of the sensitive isolate B05.10 (Zhang et al., 2020).

The criteria used for classifying the resistance level of isolates was based on their EC_50_. For fenhexamid was: sensitive (EC_50_ < 1 ppm), low resistance (EC_50_ 1 to 5 ppm), weak resistance (EC_50_ 5 to 10 ppm), moderate resistance (EC_50_ 10 to 50 ppm), and high resistance (EC_50_ > 50 ppm) (Avenot et al., 2020). For carboxin, isolates were classified as sensitive (EC_50_ < 0.94 ppm), low resistance (EC_50_ 0.95 to 1.8 ppm), and moderate to highly resistant (EC_50_ > 1.9 ppm) (Veloukas et al., 2011). For prochloraz, the EC_50_ value presented by the sensitive isolate B05.10 was used as a standard, and the degree of resistance above or below this value was determined.

### Amplification of SDHB, ERG27, and CYP51 genes

DNA from *Botrytis* isolates was used to amplify by PCR the target genes of each fungicide. The primers used for *SDHB*, *ERG27*, and *CYP51* are listed in Table 2. The reactions were carried out in a final volume of 20 μL, containing 1X buffer, 2 mM MgCl2, 0.2 mM dNTPs, 0.2 μM of each primer, 1 U of PlatinumTM Taq DNA Polymerase (Invitrogen), and 2 μL of DNA. The amplification of the *SDHB* and *ERG27* genes was performed under the following thermal profile: 95°C for 1.5 min, followed by 35 cycles of 95°C for 30 s, 60°C for 30 s, and 72°C for 1 min, with a final extension at 72°C for 5 min. For the *CYP51* gene, the amplification conditions were the following: 95°C for 1.5 min, followed by 35 cycles of 95°C for 30 s, 57°C for 30 s, and 72°C for 1.5 min, with a final extension at 72°C for 5 min.

**Table 2:** Primers used for the amplification and sequencing of the *SDHB*, *ERG27*, and *CYP51* genes.

Gene	Primer name	Sequence (sense 5′-3′)	Fragment size	Reference
*SDHB*	IpBcBeg-F	CCACTCCTCCATAATGGCTGCTCTCCGC	903 pb	Nielsen et al. (2022)
	IpBcBeg-R	CTCATCAAGCCCCCTCATGATATC		
*ERG27*	Erg27Beg	TGGGATTACCACCATGGGAGACAAGTG	1,580 pb	Cosseboom and Hu (2021)
	Erg27End	CAATGGTTCCGCATTTCTTTGCCTCCC		
*CYP51*	BcCyp51-I1F	TGCGATGGGGATTCTTGAAG	1,569 pb	Zhang et al. (2020)
	BcCyp51-I2R	TTATCGTCGCTCCCAAGCTAC		
	BcCyp51Seq-F*	CATCTACACTGCTTCGCACACTCTGC		
	BcCyp51Seq-R^*^	TATCAGATTTGAACTCTGCTTCCG		

^*^Primers used for sequencing the CYP51 gene.

The products obtained from each amplification were electrophoresed at 80 V on 1.5% agarose gel. The amplicons obtained were sequenced using the Sanger method with primers for *SDHB* and *ERG27*, as well as primers BcCyp51Seq-F and BcCyp51Seq-R for *CYP51* at Macrogen Inc., Korea (Table 2). The sequence of each amplified product was analyzed using the CLC Main Workbench v5.5 (CLCBio) program. They were then compared with reference sequences from the B05.10 isolate obtained from the NCBI database under accession number CP009805.1.

## Results

### Morphological and microscopic characterization of *Botrytis* isolates

A total of 12 monosporic isolates exhibiting macro and microscopic characteristics associated with the genus *Botrytis* were obtained. Four isolates originated from rose crops located in Madrid (MO), four isolates from rose crops located in El Rosal (ML), three isolates from rose crops located in Tocancipá (CP), and one isolate from the greenhouses of FAC (NT) (Table 3).

**Table 3:** *Botrytis* isolates obtained from the three rose-producing crops in Cundinamarca and from the greenhouses of the Faculty of Agricultural Sciences (FAC) at the Universidad Nacional de Colombia, Bogotá campus.

Crop Madrid (MO)	Crop El Rosal (ML)	Crop Tocancipá (CP)	Greenhouses FCA	Reference strain
MOP1	MLP1	CPP1	NT*	B05.10
MOP2	MLP2	CPP2		
MOP3	MLP3	CPP3		
MOP4	MLP4			

^*^NT: Non-treated isolate (from roses without fungicide application).

The macroscopic characteristics of the colonies showed morphological differences in color, appearance, sporulation, and sclerotia formation between isolates from the same rose-producing crops, between crops and in comparison, with the NT isolate and the B05.10 strain. The formation of sclerotia was the most variable trait among all analyzed isolates. However, the color and appearance of the colonies did not vary significantly among them (Table 4).

**Table 4:** Morphological, microscopical, and genetic characteristics of *Botrytis* isolates.

Morphological characteristics					Genetic changes		
Isolate	Colony color (Pantone^®^ reference)	Colony appearance	Formation of sclerotia	Spore production in microculture			
					*ERG27*	*SDHB*	*CYP51*
B05.10	Gray-Brown (401C-4715C)	Cottony	No	Yes	–	–	–
NT	Gray-Brown (401C-4715C)	Cottony	No	Yes	L195F	H272R	Y136F
MOP1	Gray-Brown (Cool Gray 1C-408C)	Cottony	Yes	Yes	L195F	H272Y	–
MOP2	Gray (Warm Gray 2C)	Cottony	Yes	Yes	–	–	–
MOP3	Gray-Brown (Cool Gray 1C-4645C)	Cottony	Yes	Yes	L195F	H272R	–
MOP4	Gray (Warm Gray 8C)	Cottony	Yes	Yes	L195F	H272R	–
MLP1	Gray-Brown (Cool Gray 1C-4655C)	Cottony	No	Yes	L400F	H272R	–
MLP2	Gray-Brown (Warm Gray 1C-Warm Gray 6C)	Cottony	Yes	Yes	L195F	H272R	Y136F
MLP3	Gray-Brown (Warm Gray 1C-Warm Gray 6C)	Cottony	Yes	Yes	–	P225F	–
MLP4	Gray-Brown (427C-Warm Gray 8C)	Cottony	Yes	No	F412S	H272Y	Y136F
CPP1	Brown (4725C)	Cottony	No	Yes	L195F	H272R	–
CPP2	Gray-Brown (Warm Gray 1C-Cool Gay 9C-465C)	Cottony	Yes	No	L195F	–	N230K
CPP3	Gray-Brown (Warm Gray 1C-465C)	Powdery	Yes	No	L400F	H272R	–

Regarding microscopic characteristics, the time for conidial formation varied based on the origin of each isolate. The NT, B05.10, and isolates from the MO crop formed conidia within 7 days of setting up the microculture. Similarly, three isolates from the ML crop (MLP1, MLP2, and MLP3) and one from the CP crop (CPP1) showed conidia formation after the same evaluation period. However, in isolates MLP4, CPP2, and CPP3, conidia presence was not evident (Table 4).

### Molecular identification and phylogenetic analysis

**Figure 1:**
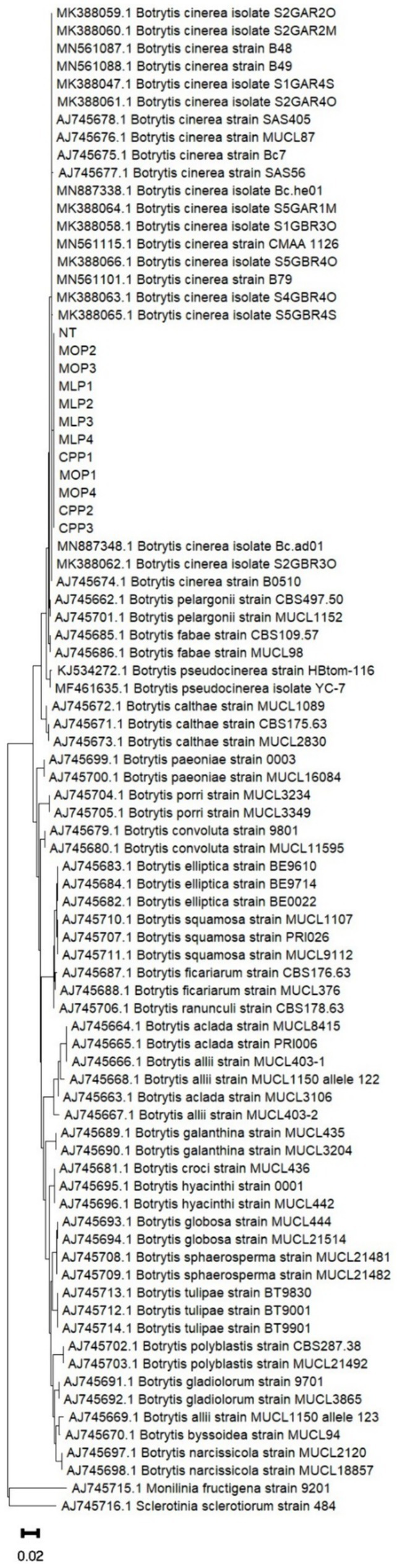
Phylogenetic tree based on concatenated sequences of the *G3PDH*, *HSP60*, and *RPB2* genes from *Botrytis* isolates obtained from rose-producing crops in the Cundinamarca department under fungicide application pressure. The method used was Maximum Likelihood, Kimura model with Gamma distribution, with 1,000 bootstrap repetitions. Sequences of the three genes from *Monilia fructigena* and *Sclerotinia sclerotiorum* were used as outgroups.

The BLASTn analysis performed on the different sequences obtained from *HSP60* gene determined the identity of all isolates as *Botrytis cinerea* within an approximate range of 99 to 100% (data not shown). Additionally, for each isolate, the sequences of the *G3PDH*, *HSP60*, and *RPB2* genes were analyzed individually and concatenated to determine the phylogenetic relationship and confirm their identity (Supplementary Table S1) and concatenation Individual analysis and concatenation of *G3PDH*, *HSP60*, and *RPB2* gene sequences revealed that all isolates from rose-producing crops (MO, ML, and CP) and NT were within the *B. cinerea* clade and formed a single group in the phylogenetic tree (Figure 1). This confirmed that all strains belong to the species *B. cinerea*.

### Sensitivity of *Botrytis cinerea* isolates to fenhexamid, carboxin, and prochloraz

Differences in sensitivity were found among *Botrytis* isolates to fenhexamid, carboxin, and prochloraz, both within and between rose-producing crops. The ranges of EC_50_ and FR values are shown in Table 5. For fenhexamid, EC_50_ values ranged from 0.02 to 4.21 ppm, 41% of the isolates were classified as sensitive, while 59% of the isolates showed low resistance to this fungicide. Regarding carboxin, 92% of the isolates exhibited moderate to high resistance (EC_50_ > 0.94 ppm), with EC_50_ ranges between 0.2 and 4.54 ppm. The NT isolate from roses without fungicide exposure showed a loss of sensitivity to both fenhexamid and carboxin compared to the reference-sensitive isolate B05.10. On the other hand, the 11 isolates collected from fungicide-exposed roses showed high sensitivity levels to prochloraz, with a mean EC_50_ value of 0.36 ppm. The lowest EC_50_ value was 0.09 ppm, observed in the MOP2 isolate, and the highest was recorded for CPP2 with 1.08 ppm. In this case, the NT isolate was more sensitive to prochloraz (EC_50_ 0.17 ppm) than B05.10 (EC_50_ 0.26 ppm).

**Table 5:** EC50 values, FR, and mutations exhibited by *Botrytis* isolates.

Fenhexamid					Carboxin				Prochloraz			
	Sensitivity			Genetics	Sensitivity			Genetics	Sensitivity			Genetics
Isolate	EC_50_ (ppm)	RL	RF	Mutations found	EC50 (ppm)	RL	RF	Mutations found	EC50 (ppm)	RL	RF	Mutations found
NT	0,56	S	28,0	L195F	3,34	MR	16,7	H272R	0,17	S	0,7	Y136F
MOP1	4,21	LR	210,5	L195F	3,21	MR	16,1	H272Y	0,23	S	0,9	–
MOP2	0,03	S	1,5	–	0,65	S	3,3	–	0,09	S	0,3	–
MOP3	0,06	S	2,0	L195F	2,94	MR	14,7	H272R	0,26	S	1,0	–
MOP4	1,14	LR	57,0	L195F	2,66	MR	13,3	H272R	0,46	LR	1,8	–
MLP1	0,62	S	31,0	L400F	2,01	MR	10,1	H272R	0,45	LR	1,7	–
MLP2	3,81	LR	190,5	L195F	3,00	MR	15,0	H272R	0,44	LR	1,7	Y136F
MLP3	0,02	S	1,0	–	4,26	MR	21,3	P225F	0,22	S	0,8	–
MLP4	3,69	LR	184,5	F412S	2,00	MR	10,0	H272Y	0,58	LR	2,2	Y136F
CPP1	1,42	LR	71,0	L195F	3,05	MR	15,3	H272R	0,20	S	0,8	–
CPP2	1,17	LR	58,5	L195F	4,54	MR	22,7	–	1,08	LR	4,2	N230K
CPP3	3,80	LR	190,0	L400F	1,93	MR	9,7	H272R	0,10	S	0,4	–
B05.10	0,02	S	1,0		0,20	S	1,0		0,26	S	1,0	

Fungicide concentration causing 50% inhibition of mycelial growth (EC_50_ values) is presented in ppm. Resistance factor (RF) values were calculated by dividing the EC_50_ value of each isolate by the EC_50_ value of the B05.10 isolate. Resistance levels (RL): S (sensitive), LR (low resistance), MR (moderate resistance). fenhexamid classification according to Avenot et al. (2020): sensitive (EC_50_ < 1 ppm), low resistance (EC_50_ 1 to 5 ppm), weakly resistant (EC_50_ 5 to 10 ppm), moderate resistant (EC_50_ 10 to 50 ppm), highly resistant (EC_50_ > 50 ppm). carboxin classified according to Veloukas et al. (2011): sensitive (EC_50_ < 0.94 ppm), low resistance (EC_50_ 0.95 to 1.8 ppm), moderate to high resistance (EC_50_ > 1.9 ppm). Resistance levels for prochloraz were determined according to EC_50_ values presented by the B05.10 isolate: sensitive (EC_50_ < 0.26 ppm), low resistance (EC_50_ > 0.26 ppm). Regarding the found mutations, the letters correspond to the amino acid, and the number corresponds to the position where the amino acid change occurred.

### Mutations in the ERG27, SDHI, and CYP51 genes of *Botrytis cinerea* isolates

Changes at nucleotide and amino acid-level were detected in the sequences of the genes related to resistance. In isolates from crops, mutations were observed in 82% of the sequences for *ERG27*. The L195F mutation was predominant in six of the eleven isolates (MOP4, MOP6, MOP7, MLP2, CPP1, and CPP2) (Table 4). Similarly, the L400F substitution was found in two cases (MLP1, CPP3). The MLP4 isolate had the F412S mutation, and MOP2 and MLP3, did not have mutations of interest. Finally, the NT isolate showed the L195F mutation for this gene.

*SDHB* sequences exhibited mutations in 90% of the isolates. The alterations found predominantly in position H272, with H272R or H272Y being the most abundant inclusive in the NT isolate (Table 4). Additionally, the MLP3 isolate showed the P225F mutation, and MOP2 and CPP2 did not have mutations in this gene.

Regarding the *CYP51* sequences, the Y136F mutation was found in three sequences, NT, MLP2, and MLP4. Similarly, the N230K mutation was found in the CPP2 isolate. All the other isolates did not exhibit mutations in this gene.

The mutations found in *ERG27*, *SDHI*, and *CYP51* genes were not associated with the differences in the morphological characteristics of the isolates evaluated in this study, such as the color and appearance of the colony, sclerotia formation, and sporulation.

## Discussion

Gray mold, caused by *Botrytis* species, stands out as one of the most significant diseases and a primary factor leading to economic losses in greenhouse flower production (Boddy, 2015; Elad et al., 2016; Yin et al., 2023). In Colombia, knowledge of this pathogen has focused on the assessment of morphological components, both for identification and evaluation of fungicides for management. However, the existing gap regarding the genetic characterization of *Botrytis* populations and the dynamics of fungicide resistance demands a deeper analysis of this fungus and its populations.

At the morphological level, traits such as sporulation and sclerotia formation were decisive characteristics for primary differentiation among the obtained isolates. It was established that some isolates originating from rose-producing crops had reduced conidia formation and preferred the development of resistance structures. This phenomenon could be explained by the fact that *Botrytis* resistance to different fungicides can alter its sporulation capacity, mycelial growth, virulence, production, and viability of sclerotia, among others, referred to as fitness cost (Veloukas et al., 2014). These elements can provide advantages or disadvantages for the survival, growth, and reproduction of the pathogen under certain conditions, including stress due to fungicide selection (Chen et al., 2013; Zhang et al., 2020). However, no direct relationship was found between the morphological characteristics and mutations in the target genes for each fungicide. For example, isolate B05.10 (a laboratory wild strain, without fungicide pressure selection and alterations at the molecular level) showed similar morphological behavior to NT (field isolate without fungicide exposure with mutations in the three genes evaluated *ERG27*, *SDHB*, *CYP51*). Likewise, there was no correlation between the morphological pattern of the isolates exposed to fungicides and the morphological characteristics of the NT isolate. Even so, we found differences in the formation of reproductive or resistant structures, among the isolates recovered from rose crops, suggesting that populations of *B. cinerea* with resistance to fungicides could spread despite a certain fitness cost not related with the mutations detected. Additionally, this represents a risk for gray mold management, as the progeny of isolates like these may constitute a higher percentage of the population in subsequent generations (Yin et al., 2023). This implies considering the use of evaluated compounds.

In this study, molecular markers (*HSP60*, *G3PDH*, and *RPB2*) proposed by Staats et al. (2005, 2007) were used to investigate the genetic relationships and diversity among *Botrytis* species in rose crops. Our results confirmed that *B. cinerea* is the causal agent of gray mold in rose-producing crops of the Cundinamarca department. The use of powerful phylogenetic analysis tools represents an upgrade and a more precise method in contrast to classical microbiologic and biochemical methods (Hyde et al., 2014; Garfinkel, 2021). These analyses showed a coherent and consistent clustering of all sequences within this species, confirming the precise and reliable identification of rose isolates from rose-producing crops in Cundinamarca. In the analysis of the *RPB2* gene, similarities were determined among rose isolates that grouped them into the same cluster. However, they were separated from other reference *Botrytis* species sequences. This led to distinguishing the sequences of isolates subjected to frequent fungicide management as a particular population, phylogenetically distant from other *B. cinerea* strains like Bc7, MUCL87, SAS405, SAS56, and B05.10, recovered from hosts such as grapevines (*Vitis vinifera*) in Italy or the Netherlands (Staats et al., 2005). Although none of the sequences used as a reference came from rose crops, for instance, our findings can serve as a starting point for future studies on *B. cinerea* populations on this host. The *RPB2* gene encodes a protein with a low evolutionary change rate, used to understand phylogenetic relationships at inter- and intra-specific levels in various fungi (Malkus et al., 2006). It has also been a molecular marker providing additional information related to specific adaptation or evolution characteristics (Staats et al., 2007; Garfinkel, 2021). The evidence presented in the *RBP2* gene analysis affirms that *B. cinerea* in rose crops in Colombia has a genetic identity classifying them as a unique and specific population. This is the first report of these characteristics in *B. cinerea* from rose crops in Colombia.

The analysis of fungicide resistance evidenced specific mutations in the genes related to resistance of fenhexamid, carboxin, and prochloraz. For fenhexamid, some crop isolates showed low resistance and sensibility according to Avenot et al. (2020). Additionally, the control strains NT and B05.10 were sensible to this fungicide. At the molecular level, some target sites showed changes in the *ERG27* gene sequences. One of the predominant changes occurred in the Leucine at positions 195 and 400 of the open reading frame, which was found even in the NT isolate. According to Fillinger et al. (2008), strains with amino acid changes between positions 195 and 400 of the protein are categorized as HydR3^−^, which indicates moderate to slight resistance to fenhexamid and is associated with the presence of the *erg27^HdR3-^* allele. For example, the L195F mutation has been reported in *Botrytis* spp. isolates from French and German vineyards with weak to moderate resistance to fenhexamid. Additionally, this mutation was easily detected in other field populations, even before the introduction of this compound to the market (Fillinger et al., 2008). Therefore, the L195F mutation is not directly linked to fungicide resistance and may occur in strains with or without fungicide pressure. However, despite the description of *Botrytis* spp. resistance to fenhexamid worldwide (Albertini and Leroux, 2004; Esterio et al., 2007; Fillinger et al., 2008; Avenot et al., 2020), we found the L195F mutation in 58% of the analyzed isolates from rose crops in Colombia, with represent data that has not been previously reported in local *Botrytis* strains. Furthermore, we found the F412S mutation in one of the analyzed isolates (MLP45). This Phenylalanine-to-Serine substitution at codon 412 of the *ERG27* gene was classified as responsible for high resistance to fenhexamid. However, the low proportion found of this mutation in our analyses may be due to a fitness cost of these strains (Fillinger et al., 2008; Leroux et al., 2010).

For carboxin, we found low sensitivity to this fungicide, even in the untreated isolate (NT). At the molecular level, mutations were detected in the codon 272 of the *SDHB* gene, where the amino acid histidine (H) was replaced by tyrosine (H272Y) or arginine (H272R) in 75% of cases, and in a minor proportion, proline was replaced by phenylalanine in codon 225 (P225F). These mutations have been associated with *B. cinerea* resistance to this fungicide (Leroux, 2007; Amiri et al., 2014; De Miccolis Angelini et al., 2014). However, the carboxin resistance has been also associated with multiple mutations in another succinate dehydrogenase (SDH) enzyme subunit. Amiri et al. (2020) demonstrated that mutations in the SDH subunit C (SDHC) in *B. cinerea* isolates, with or without mutations in the SDHB subunit, generated changes in susceptibility to SDHI fungicides as well as changes in morphological characteristics. Although mutations in *SDHC* were not evaluated in this study, differences in morphology and resistance levels between the isolates and NT could be associated with the presence or absence of changes in both succinate dehydrogenase subunits (Amiri et al., 2020). However, *Botrytis* has shown moderate resistance not only to carboxin but also to other molecules with the same mechanism of action, such as Boscalid or Isopyrazam, and hypersensitivity to Fluopyram (Bardas et al., 2010; Lalève et al., 2014), all of them available for gray mold management in Colombia. Mutations have also been detected in *Botrytis* strains without exposure to fungicides (Shao et al., 2021).as we observed in our NT isolate. A possible explanation for this is that NT could have originated from an isolate previously exposed to this fungicide, and through mechanisms of migration of *B. cinerea* spores by wind or water or through human and animal mediated transmission mechanisms, facilitated the establishment of the fungus in a rose crop or garden that had not previously been treated with fungicides, as evidenced in native plant crops where the disease had not previously been reported (Notte et al., 2021). Therefore, there is a need to replace these compounds for gray mold management or their rational use, using as reference the phenotypic and genetic characterization.

Similarly, all tested isolates were sensitive to the fungicide prochloraz. 60% of these showed higher sensitivity than the reference strain B05.10. Unlike with the other fungicides in this study, the untreated isolate (NT) from untreated roses exhibited high sensitivity to this molecule. Tyrosine (Y) to Phenylalanine (F) substitutions at position 136 (Y136F) and asparagine (N) to lysine (K) at position 230 (N230K) were found in the *CYP51* gene sequences. Alterations in this gene have been reported as a common mechanism of fungal resistance to these compounds (Wyand and Brown, 2005), and several mutations in addition to those found have been associated with resistance to this molecule, such as Y136F, V136A, I381V, D134G, and S524T (Cools et al., 2013). However, although mutations were found in some isolates, all showed an *in vitro* inhibition on growth under the presence of prochloraz, indicating that the fungicide remains effective in *Botrytis* control, although the isolates have the mutations Y136F and N230K. Therefore, the data obtained in this study can be useful for monitoring possible changes in the sensitivity of populations of this fungus in ornamental crops, and the mean of 0.22 ppm of prochloraz given by the EC_50_ of the reference strain B05.10 and the untreated isolate (NT) could be considered as a sensitivity parameter for future evaluations of this fungicide.

Previous studies used molecular analysis to understand the role of *B. cinerea* as the causal agent of gray mold in ornamental crops in Colombia and their resistance profiles to fungicides (Muñoz et al., 2019). Despite this, our work complements and deepens the characterization of *Botrytis* isolates from Colombian rose crops boarding phenotypic and molecular characterization and evaluating mechanism of resistance at the molecular level, searching mutations in target genes. Fungicide treatments are essential to maintain healthy crops and achieve reliable and high-quality yields (Yin et al., 2023), and both detection and monitoring of resistance in *Botrytis* populations are crucial for implementing appropriate resistance management strategies to select the most effective fungicides in disease control (Richards et al., 2021; Yin et al., 2023). Our results demonstrate that *Botrytis* populations in Colombian crops present a unique identity associated with the same *B. cinerea* specie. Additionally, they show different levels of sensitivity and mutations in the target sites of each fungicide, sometimes simultaneously. These findings highlight the importance of understanding the persistence of resistant *B. cinerea* populations on fields, the effect of fungicide rotation programs on mutant selection, and the rational use of fungicides. It is essential to understand the dynamics of resistance and its evolution in *Botrytis* populations, as well as to extend this evaluation to other molecules used in the field. This will enable proper management of chemical control and delay the onset of resistance on Colombian floriculture.

## Data availability statement

Data has been supported in the following link: https://doi.org/10.6084/m9.figshare.26195603.

## Author contributions

DG: Conceptualization, Data curation, Formal analysis, Investigation, Methodology, Writing – original draft, Writing – review & editing. CS: Investigation, Methodology, Writing – review & editing. HG: Formal analysis, Resources, Writing – review & editing. ML: Data curation, Formal analysis, Investigation, Methodology, Resources, Writing – review & editing. AG: Conceptualization, Data curation, Formal analysis, Investigation, Methodology, Supervision, Writing – review & editing.

## Footnotes

^1^https://www.pantone-colours.com

^2^https://blast.ncbi.nlm.nih.gov/Blast.cgi
