# Supplementary material for: Genotypic and phenotypic characterization of resistance to fenhexamid, carboxin, and, prochloraz, in Botrytis cinerea isolates collected from cut roses in Colombia
Source: Front Microbiol. 2024 Jul 31;15:1378597. doi: 10.3389/fmicb.2024.1378597 (PMC11323744; doi:10.3389/fmicb.2024.1378597)
Supplement: Supplementary file 1 [file Data_Sheet_1.PDF]

Table S1. GenBank accession numbers of *RPB2, HSP60,* and *G3PDH* genes used for phylogenetic analysis.

			GenBank Accession Number sequences		
**Species**	**Collection Number**	**Geographic Origin**	***RPB2***	***HSP60***	***G3PDH***
B. aclada	PRI006	—	AJ745665	AJ716051	AJ704993
B. aclada	MUCL3106	USA	AJ745663	AJ716049	AJ704991
B. aclada	MUCL8415	Germany	AJ745664	AJ716050	AJ704992
B. alli	MUCL403	The Netherlands	AJ745666 (allele 1)	AJ716055 (allele 1)	AJ704996 (allele 1)
			AJ745667 (allele 2)	AJ716056 (allele 2)	AJ704997 (allele 2)
				AJ716057 (allele 3)	
				AJ716058 (allele 4)	
B. alli	MUCL1150	Norway	AJ745668 (allele 1)	AJ716052 (allele 1)	AJ704994 (allele 2)
			AJ745669 (allele 2)	AJ716053 (allele 2)	AJ704995 (allele 1)
				AJ716054 (allele 3)	
B. byssoidea	MUCL94	USA	AJ745670	AJ716059	AJ704998
B. calthae	CBS 175.63	USA	AJ745671	AJ716060	AJ704999
B. calthae	MUCL1089	Bélgica	AJ745672	AJ716061	AJ705000
B. calthae	MUCL2830	Belgium	AJ745673	AJ716062	AJ705001
B. convoluta	9801	The Netherlands, Lisse	AJ745679	AJ716068	AJ705007
B. convoluta	MUCL11595	USA	AJ745680	AJ716069	AJ705008
B. cinerea	SAS56	Italy	AJ745677	AJ716067	AJ705006

B. cinerea	SAS405	Italy	AJ745678	AJ716066	AJ705005
B. cinerea	B05.10	—	AJ745674	AJ716063	AJ705002
B. cinerea	BC7	The Netherlands	AJ745675	AJ716064	AJ705003
B. cinerea	NT	Colombia	PP737772	PP737760	PP737748
B. cinerea	MOP1	Colombia	PP737773	PP737761	PP737749
B. cinerea	MOP2	Colombia	PP737774	PP737762	PP737750
B. cinerea	MOP3	Colombia	PP737775	PP737763	PP737751
B. cinerea	MOP4	Colombia	PP737776	PP737764	PP737752
B. cinerea	MLP1	Colombia	PP737777	PP737765	PP737753
B. cinerea	MLP2	Colombia	PP737778	PP737766	PP737754
B. cinerea	MLP3	Colombia	PP737779	PP737767	PP737755
B. cinerea	MLP4	Colombia	PP737780	PP737768	PP737756
B. cinerea	CPP1	Colombia	PP737781	PP737769	PP737757
B. cinerea	CPP2	Colombia	PP737782	PP737770	PP737758
B. cinerea	CPP3	Colombia	PP737783	PP737771	PP737759
B. croci	MUCL436	The Netherlands	AJ745681	AJ716070	AJ705009
B. elliptica	BE9714	The Netherlands, Elsloo	AJ745684	AJ716073	AJ705012
B. elliptica	BE9610	The Netherlands	AJ745683	AJ716072	AJ705011
B. elliptica	BE0022	The Netherlands, Smilde	AJ745682	AJ716071	AJ705010
B. fabae	CBS 109.57	The Netherlands	AJ745685	AJ716074	AJ705013
B. fabae	MUCL98	Spain	AJ745686	AJ716075	AJ705014
B. ficariarum	CBS 176.63	Belgium	AJ745687	AJ716076	AJ705015
B. ficariarum	MUCL376	Belgium	AJ745688	AJ716077	AJ705016
B. galantina	MUCL435	The Netherlands	AJ745689	AJ716079	AJ705018
B. galantina	MUCL3204	The Netherlands	AJ745690	AJ716078	AJ705017
B. gladiolorum	9701	—	AJ745691	AJ716080	AJ705019
B. gladiolorum	MUCL3865	The Netherlands	AJ745692	AJ716081	AJ705020
B. globosa	MUCL444	Belgium	AJ745693	AJ716083	AJ705022
B. globosa	MUCL21514	UK	AJ745694	AJ716082	AJ705021
B. hyacinthi	0001.	The Netherlands, Lisse	AJ745695	AJ716084	AJ705023

B. hyacinthi	MUCL442	The Netherlands, Breezand	AJ745696	AJ716085	AJ705024
B. narcissicola	MUCL18857	UK	AJ745698	AJ716086	AJ705025
B. narcissicola	MUCL2120	Canada	AJ745697	AJ716087	AJ705026
B. paeoniae	MUCL16084	Belgium	AJ745700	AJ716089	AJ705028
B. paeoniae	0003.	The Netherlands	AJ745699	AJ716088	AJ705027
B. pelargonii	CBS 497.50	Norway	AJ745662	AJ716046	AJ704990
B. pelargonii	MUCL1152	Norway	AJ745701	AJ716090	AJ705029
B. polyblastis	MUCL21492	UK	AJ745703	AJ716092	AJ705031
B. polyblastis	CBS287.38	UK	AJ745702	AJ716091	AJ705030
B. porri	MUCL3234	—	AJ745704	AJ716093	AJ705032
B. porri	MUCL3349	Belgium	AJ745705	AJ716094	AJ705033
B. ranunculi	CBS178.63	USA	AJ745706	AJ716095	AJ705034
B. sphaerosperma	MUCL21481	UK	AJ745708	AJ716096	AJ705035
B. sphaerosperma	MUCL21482	UK	AJ745709	AJ716097	AJ705036
B. squamosa	PRI026	—	AJ745707	AJ716100	AJ705039
B. squamosa	MUCL1107	USA	AJ745710	AJ716098	AJ705037
B. squamosa	MUCL9112	The Netherlands	AJ745711	AJ716099	AJ705038
B. tulipae	BT9830	The Netherlands	AJ745713	AJ716102	AJ705041
B. tulipae	BT9001	The Netherlands	AJ745712	AJ716101	AJ705040
B. tulipae	BT9901	The Netherlands	AJ745714	AJ716103	AJ705042
M. fructigena	9201	—	AJ745715	AJ716047	AJ705043
S. sclerotiorum	484I	—	AJ745716	AJ716048	AJ705044
